# Learning to maximize reward rate: a model based on semi-Markov decision processes

**DOI:** 10.3389/fnins.2014.00101

**Published:** 2014-05-23

**Authors:** Arash Khodadadi, Pegah Fakhari, Jerome R. Busemeyer

**Affiliations:** Department of Psychological and Brain Sciences, Indiana UniversityBloomington, IN, USA

**Keywords:** semi-Markov decision process, average reward rate maximization, speed-accuracy trade-off, reinforcement learning, sequential sampling models, diffusion process, decision threshold

## Abstract

When animals have to make a number of decisions during a limited time interval, they face a fundamental problem: how much time they should spend on each decision in order to achieve the maximum possible total outcome. Deliberating more on one decision usually leads to more outcome but less time will remain for other decisions. In the framework of sequential sampling models, the question is how animals learn to set their decision threshold such that the total expected outcome achieved during a limited time is maximized. The aim of this paper is to provide a theoretical framework for answering this question. To this end, we consider an experimental design in which each trial can come from one of the several possible “conditions.” A condition specifies the difficulty of the trial, the reward, the penalty and so on. We show that to maximize the expected reward during a limited time, the subject should set a separate value of decision threshold for each condition. We propose a model of learning the optimal value of decision thresholds based on the theory of semi-Markov decision processes (SMDP). In our model, the experimental environment is modeled as an SMDP with each “condition” being a “state” and the value of decision thresholds being the “actions” taken in those states. The problem of finding the optimal decision thresholds then is cast as the stochastic optimal control problem of taking actions in each state in the corresponding SMDP such that the average reward rate is maximized. Our model utilizes a biologically plausible learning algorithm to solve this problem. The simulation results show that at the beginning of learning the model choses high values of decision threshold which lead to sub-optimal performance. With experience, however, the model learns to lower the value of decision thresholds till finally it finds the optimal values.

## 1. Introduction

In many problems that animals and humans encounter, the quality of a desired outcome that they can achieve depends on the amount of a resource they spent. For example, one can pay more money (resource) to buy a more stylish (higher quality) coat (desired outcome). If the resource is limited (which is almost always the case), the animal or human should decide how much of the resource she is willing to spend on obtaining one outcome. By spending more of the resource on an outcome the quality increases but less would be left for other outcomes. A rational animal or human, then, should decide how to allocate the resource for obtaining each outcome to maximize the total amount of obtained outcome. That is, she should find out what resource allocation maximizes *outcome per unit of the resource*.

One interesting example of a situation in which the subject should trade a resource with the quality of the outcome is perceptual decision making in which the subject should detect a noisy stimulus and choose a proper response based on it. Because of the noise in the stimulus, to make more accurate responses the subject should spend more time to detect the stimulus. Since faster responses are less accurate, the subject should trade between the amount of time (resource) and the accuracy (which determines the quality of the outcome). This leads to the so-called speed-accuracy tradeoff (SAT).

In the past few decades, computational modeling has been a popular method for investigating the mechanisms underlying perceptual decision making. A large class of models of perceptual decision making, called sequential sampling models, assume that the subject sequentially samples from the stimulus (Link and Heath, 1975; Townsend and Ashby, 1983; Luce, 1986; Smith and Vickers, 1988; Busemeyer and Townsend, 1993; Smith, 2000; Usher and McClelland, 2001; Ratcliff and Smith, 2004). These samples are noisy and so the decision cannot be made based on a single sample. These models propose that the subject responds whenever the accumulated evidence favoring one of the responses exceeds a specific value called the *decision threshold*. This way, these models separate the perceptual process from the decisional process. The evidence accumulation models the perceptual process and is assumed to be affected by the physical stimulus. The decisional process is modeled by the decision threshold and is assumed to be controlled by the subject. Higher values of the decision threshold mean that more information is needed for making a decision and so the decisions will be more accurate. However, accumulating more information takes more time and so decisions will be slower. Thus, the SAT is explained in sequential sampling models by changes in the decision threshold. This feature of sequential sampling models has motivated a large body of research on the SAT phenomena. A standard experimental method of investigating this phenomena is to vary the emphasis on speed or accuracy in the task instructions. Sequential sampling models predict that the subjects will choose lower decision threshold in the speed condition in comparison to the accuracy conditions. This prediction has been confirmed in many studies (Ratcliff, 1978; Luce, 1986; Ratcliff, 2002; Palmer et al., 2005; Ratcliff and McKoon, 2007; Ivanoff et al., 2008; Wagenmakers et al., 2008; Bogacz et al., 2010; Forstmann et al., 2010).

Although these results show that subjects choose different values of decision threshold in response to varying the task's instructions, they do not specify what value of the decision threshold should be chosen in each condition. In other words, the results of theses studies do not provide a normative account of the SAT phenomena. The rationality notion explained above, however, suggests a possible way to provide such an explanation: if the total time of the task is fixed, a rational subject should balance between her speed and accuracy such that the total outcome obtained during the whole task is maximized. Spending more time on one trial results in less remaining time for the other trials, meaning the subject experiences fewer trials in the task. However, by spending more time on one trial the subject can increase the chance of responding correctly.

This experimental design was first suggested by Gold and Shadlen (2002). They considered a perceptual decision making task in which the total time of the task is fixed and so the total number of trials that the subject can experience depends on the average time she spends on each trial. Also, the subject receives a reward after each correct response and a penalty after each incorrect response. They proposed that a rational subject sets her decision threshold such that the expected total outcome (sum of rewards and penalties) would be maximized. Because the total time of the task is limited and fixed, this is equivalent to maximizing the expected outcome per unit time, or the *average reward rate*.

Bogacz et al. (2006) further investigated the properties of the average reward rate as a function of the task parameters (e.g., reward, penalty, stimulus salience and so on) and the parameters of a class of sequential sampling models. Specifically, they derived the relationship between the task parameters and the optimal value of the decision threshold in the experimental design of Gold and Shadlen. More recently, Simen et al. (2009) and Balci et al. (2011) conducted a series of experiments to see if human subjects can achieve the optimal performance in this experimental design. The results of these studies showed that after extensive training in tasks similar to what was proposed by Gold and Shadlen (2002), human subjects could learn to set the decision threshold at values close to optimal.

Knowing that subjects can learn to behave optimally, the next question would be how the brain learns the optimal threshold. The aim of this paper is to propose a computational framework to answer this question. To this end, we consider a more general experimental design than the design of Gold and Shadlen. In this design, instead of having one condition, trials in a block can come from one of several possible conditions and so the subject should set different decision thresholds for different conditions to achieve the maximum average reward rate (section 2). We then show that this experiment can be modeled as a stochastic process, specifically a *semi-Markov decision process* (section 4). Learning the optimal decision threshold will be framed as an optimal control problem in this stochastic environment. We then propose a biologically plausible model that can solve this problem (section 5). In the final section of this paper, we test the performance of our model in learning the optimal value of the decision threshold in different experiments (section 6).

## 2. Computational methods

Our model is developed to account for a more general experimental design than what was used in previous research on optimal SAT. To the best of our knowledge, Simen et al. (2009) conducted the first experimental study to investigate if human subjects can learn the optimal value of the decision threshold. To contrast their experimental design with the one that is considered in this paper, here we briefly explain experiment 1 of Simen et al. (2009).

The stimulus in each trial of this experiment was the well-known random-dot kinematogram. This stimulus consists of a number of dots, some of them moving coherently toward the left or toward the right, while other dots move randomly. The subjects' task is to decide in each trial if the net direction of motion is toward the left or right. The salience of the stimulus is determined by the percentage of dots that are moving coherently. Other task parameters were the reward that the subject receives after each correct response and the response-stimulus interval (RSI), the time between subject's response and the presentation of the next stimulus. Each session of the experiment consisted of 12 blocks (the number of blocks was more than 12, but here we just consider those that are relevant to our explanation). The blocks' duration was fixed (4 min) and so the number of trials in each block depended on how much time the subject spent on each trial.

Based on Gold and Shadlen's hypothesis (Gold and Shadlen, 2002), because the blocks' duration is fixed, a rational subject will try to balance her speed and accuracy such that the average reward rate is maximized. Since the total reward is the sum of the reward for each block, maximizing the total average reward rate is equivalent to maximizing it in each individual block. In experiment 1 of Simen et al. (2009), the stimulus salience and reward were held constant. RSI was held constant within each block, but manipulated across blocks. Clearly, the average reward rate is a function of RSI, since the longer the delay between the trials, the fewer trials can be experienced within a block. In addition, (Bogacz et al., 2006) showed that if the subjects' performance in this experiment is modeled in the sequential sampling framework, the optimal value of the decision threshold is a function of RSI. Therefore, to achieve the optimal performance in each block (and so maximize the total average reward rate) subjects have to set different decision thresholds for different blocks, dependent on the RSI.

Although the optimal value of the decision threshold in a block depends on the RSI in that block, it does not depend on the RSI in other blocks. In other words, to maximize the average reward rate in one block, the subject does not need to know what are the values of RSI in other blocks. Therefore, the subject can set the value of the decision threshold in each block with a specific value of RSI, independent of other blocks with different RSIs. This is the main difference between this design and the design we consider in the current paper.

**Figure 1:**
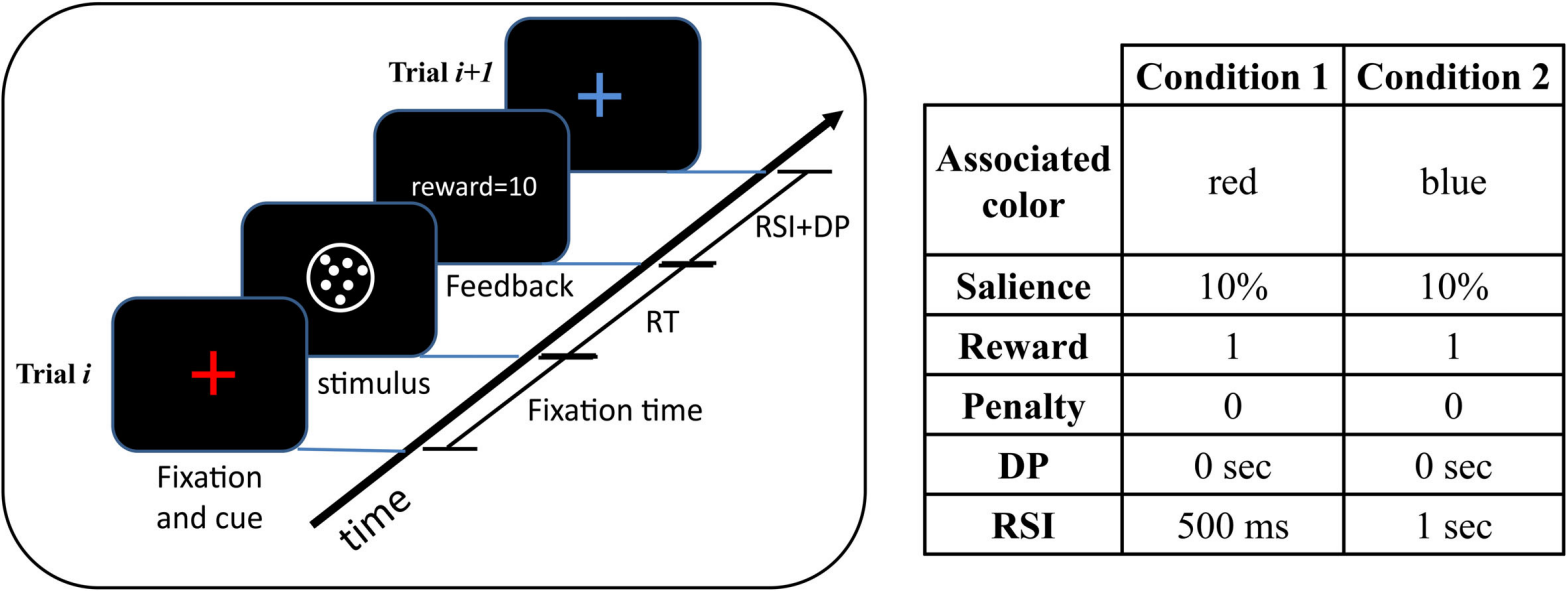
**An example of the experimental design**. In this example, each trial can come from one of two conditions with equal probability. A colored cross-hair presented at the beginning of each trial indicates the condition of the upcoming trial. After the presentation of the cue, the stimulus appears and remains on the screen till the subject responds. After responding, the subject receives feedback. The time between subject's response and the beginning of the next trial is determined by the delay penalty (DP) and response-stimulus interval (RSI). The table on the right of the figure shows the cue-condition association along with the task parameters in each condition. As seen, the two conditions differ only in the value of the RSI. See Table 1 for a description of the task parameters. In this table, a separate parameter for the fixation time is not considered and instead it is considered as a part of the RSI.

Here, we consider a more general design in which to achieve the optimal performance the subject should consider all conditions together and the optimal decision threshold for one condition depends on all other conditions in the task. As an example, consider two conditions: RSI = 500 ms and RSI = 1000 ms. In the previous design, there would be two types of blocks: in one type the RSI of all trials is 500 ms while the RSI of the trials in the other type of blocks is 1000 ms. In our design, however, trials with RSI = 500 ms and RSI = 1000 ms are all intermixed. In other words, there is no manipulation across blocks. Crucially, a cue associated with each RSI value is presented at the beginning of each trial. For example, in the task set-up shown in Figure 1, in trials with RSI = 500 ms a red cross-hair is presented as the cue while in trials with RSI = 1000 the cue is a blue cross-hair. As seen in this figure, the cue is followed by the random dots stimulus. The blocks' duration is fixed and so a rational subject should maximize the average reward rate.

Since a cue associated with the RSI of the trial is presented before the presentation of the stimulus, we assume that subjects can set different values of decision threshold for each value of RSI. In other words, the subject can associate different value of decision threshold to each cue. Thus, like the design in Simen et al. (2009), the average reward rate will be a function of the two decision thresholds. The crucial difference is that in their design the two decision thresholds are independent of each other (since RSI is only manipulated across blocks), whereas in our design the optimal threshold for one value of RSI depends on the value of the other RSI.

The reason for this dependency in our design can be conceived intuitively by noting that because the blocks' duration is fixed, every second that the subject spends on one trial, she is actually losing the opportunity to spend that time on other trials. If the other trials on average lead to higher reward, it is better to spend less time on the current trial. By being faster in one type of trials, the accuracy decreases and so the subject will lose more rewards in those trials. However, if other types of trials are “rewarding enough” it may be worth it to be fast and inaccurate in those trials which lead to less reward. This means that to set the decision threshold in each condition, the subject should consider all other conditions in the task. In the next section, we derive a formal expression of the average reward rate in our design and investigate its properties in more detail.

## 3. Average reward rate

In this section, we investigate the properties of the average reward rate as a function of the task parameters. We first state the formula for the average reward rate in the experimental design explained above. To see how this function is related to the decision threshold in different conditions, we then explain a variant of sequential sampling models called *independent race model* and show how the decision making process is modeled in this framework. Finally, we see some examples of the average reward rate for different task parameters.

### 3.1. Average reward rate as a function of task parameters

In section 2, we explained the experimental design with an example in which only one task parameter (RSI) was manipulated. Before deriving the formula for the average reward rate, we should explain the experimental design in more detail.

In the experimental design, there are several blocks with a fixed duration. Each trial in each block comes from one of the *N*_*c*_ possible “conditions” with each condition specifying the task parameters in that trial. Each trial is drawn from a given condition *C*_*i*_ with probability *P*_*i*_. As explained before, a cue presented at the beginning of each trial indicates which condition this trial is coming from. For example, in Figure 1 there are two conditions and each trial can come from one of them with equal probability. In this figure, all task parameters are the same in these two conditions except the RSI.

The subject receives a reward after each correct response and a penalty after incorrect responses. Also, there is a delay penalty after each incorrect response. This is the time that the subject should wait in addition to the RSI when the response is incorrect. The task parameters and their notations are specified in Table 1.

**Table 1:** **Task parameters in the experiment**.

**Parameter**	**Description**
*N*_*c*_	Number of conditions
*P*_*i*_	Probability of condition *i* happening
*P*^*C*^_*i*_	Probability of being correct in condition *i*
*r*^*C*^_*i*_	Reward in condition *i*
*r*^*I*^_*i*_	Penalty in condition *i*
*T*^*C*^_*i*_	Mean correct reaction time in condition *i*
*T*^*I*^_*i*_	Mean incorrect reaction time in condition *i*
*T*^*DP*^_*i*_	Delay penalty after incorrect responses in condition *i*
*T*^*RSI*^_*i*_	Response-stimulus interval in condition *i*
*T*^*ND*^	Non-decision time which is assumed to be independent of the condition

The average reward rate is defined as the average reward divided by the average time that it takes to obtain the reward. In our experimental design, since the subject can choose different decision thresholds for different conditions, the average time and average reward will be different in different conditions. The average reward in the task, then, is the weighted sum of the average reward in each condition with the weights being the probability of each condition presented in the task. The average time is computed in the same way. The average reward rate, then, can be expressed as follows:

(1) $$\bar{R}=\frac{\sum_{i=1}^{N_{c}}P_{i}\cdot \text{​}\left[r_{i}^{C}\cdot P_{i}^{C}+r_{i}^{I}\cdot (1-P_{i}^{C})\right]}{\sum_{i=1}^{N_{c}}P_{i}\cdot \text{​}\left[{\bar{T}}_{i}^{C}\cdot P_{i}^{C}+\text{​}\left({\bar{T}}_{i}^{I}+T_{i}^{DP}\right)\text{​}\cdot \left(1-P_{i}^{C}\right)\text{​}+T_{i}^{RSI}+T^{ND}\right]}$$

Among all these parameters, the subject can only control the probability of correct *P*^*i*^_*c*_, mean correct reaction time *T*^*C*^_*i*_ and mean incorrect reaction time *T*^*I*^_*i*_ in each condition *i*, by adjusting her decision threshold in each condition. All other parameters are controlled by the experimenter. The sequential sampling models specify the relationship between the decision threshold and mean reaction time and probability correct. In the next section we explain this relationship.

### 3.2. Diffusion process model of perceptual decision making

As explained before, the sequential sampling models assume that the subject accumulates noisy information favoring each response and she will respond as soon as the evidence favoring one of the responses reaches a decision threshold. Several models have been proposed based on different assumptions about the accumulation process and the decision process (see Ratcliff and Smith, 2004 for a comprehensive review of different sequential sampling models). Although different models make different predictions about a subject's performance, most of these models can fulfil the purpose of this paper. In this paper, we consider an independent race model in which the information favoring each response is accumulated in a separate accumulator. This model assumes that the subject responds as soon as the accumulated information in one of the accumulators reaches its decision threshold. The accumulated information in each accumulator is modeled as a diffusion process. In this model, the information is sampled and accumulated in continuous time. A diffusion process *X* is specified by the stochastic differential equation:

(2) dX=μ·dt+σ·dB

The parameter μ is called the drift coefficient and determines the mean of the process *X* (It can be shown that E[X(t)] = μ · *t* (see for example Smith, 2000)). This parameter is assumed to be proportional to the stimulus salience. The parameter σ is the diffusion coefficient and specifies the amount of noise in the samples. The process *dB* specifies the increments of a zero-mean Gaussian process.

**Figure 2:**
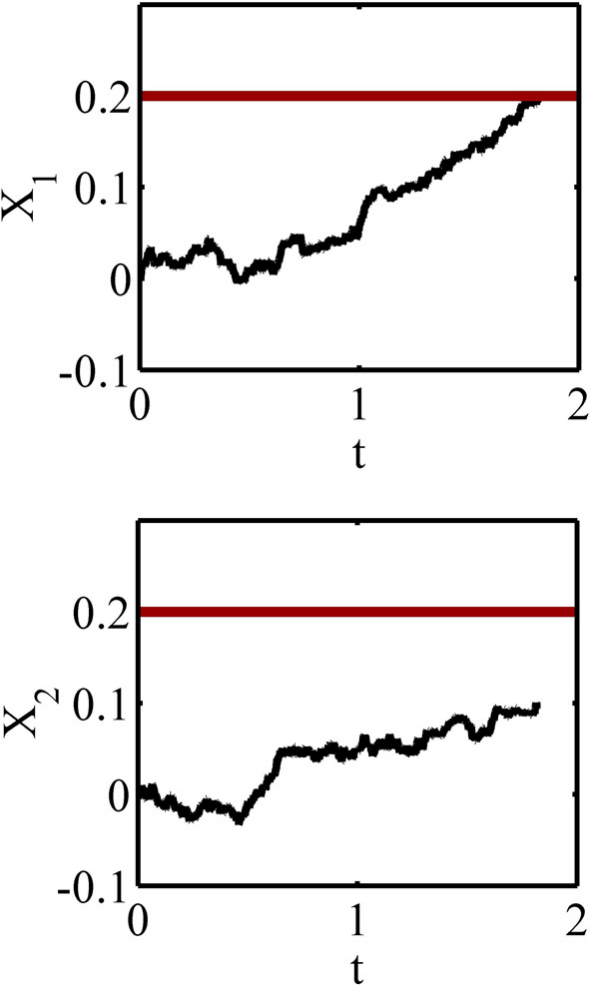
**A sample path of the accumulated information in the two accumulators of the race model**. The black paths are the accumulated information and the thick red lines are the decision thresholds. The accumulated information in the accumulator 1 reaches the decision threshold at about 1.8 s and before the accumulator 2 and so the response 1 will be selected.

Consider the random-dot kinematogram task explained in section 2. The corresponding independent race model of this task consists of two accumulators that one of them accumulates information favoring the “right” response while the other accumulates information favoring the “left” response. Each of these accumulators is a diffusion process with one decision threshold (see Figure 2). Thus, the parameters of the model are the drift coefficients μ_*i*_, the diffusion coefficients σ_*i*_ and the decision thresholds *a*_*i*_, where the subscript *i* = 1, 2 denotes the *i*^th^ accumulator. For sake of simplicity, we assume that σ_1_ = σ_2_ = σ and *a*_1_ = *a*_2_ = *a*. In this paper, we do not distinguish between the right and left responses, and instead assume that accumulator 1 corresponds to the correct response and accumulator 2 corresponds to the incorrect response. The probability of giving a correct response, as well as the probability density functions for the correct and incorrect reaction times, are expressed in Supplementary Material. These functions specify the relationship between the average reward rate function in Equation1 and the decision thresholds in the different conditions, and so all parameters being fixed, one can plot *R* as a function of the decision thresholds. Several examples are investigated in the next section.

### 3.3. Some examples

**Figure 3:**
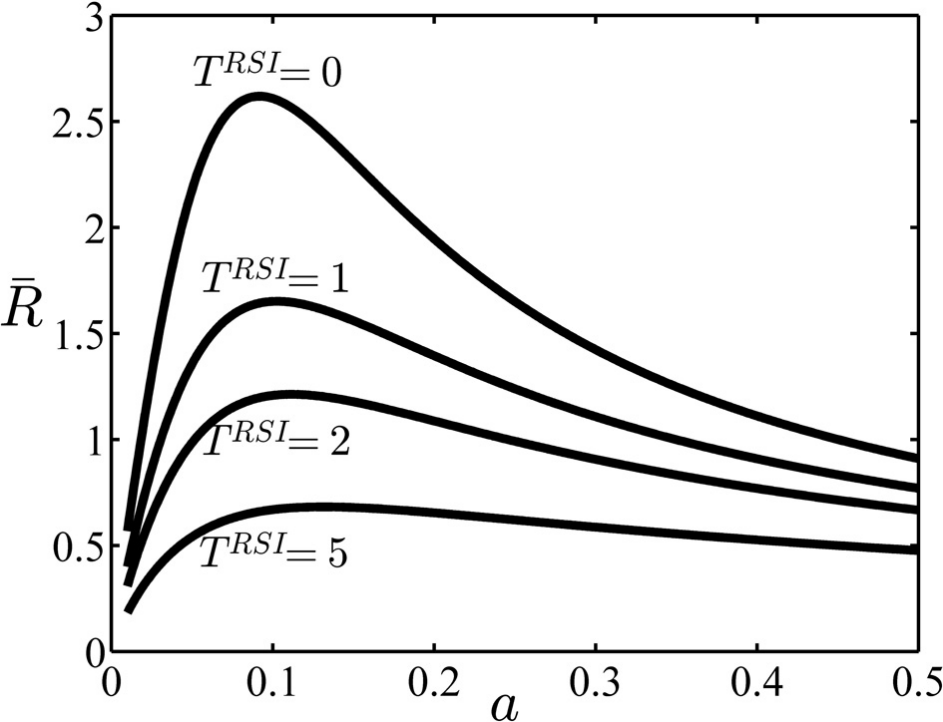
**The average reward rate in example 1**. In this example, no cue is presented at the beginning of trials and so it is assumed that the subject sets one decision threshold for all trials. In this figure, the average reward rate is plotted as a function of this decision threshold, (denoted as *a* in the figure), and for different values of the parameter *T*^*RSI*^. Other parameters are given in Table 2.

In this section, we investigate the properties of the average reward rate function in Equation1 with three examples. In the first example, we consider an experiment similar to the experimental design used previously (Simen et al., 2009; Balci et al., 2011). As explained above, in this case the subject has to set only one decision threshold for each block. The average reward rate *R* as a function of the decision threshold for the task parameters given in Table 2 and different values of *T*^*RSI*^ is shown in Figure 3. As can be seen in this figure, for all values of *T*^*RSI*^ there is one value of the decision threshold *a* that maximizes the average reward rate. The properties of the average reward rate function for another sequential sampling model, called the drift diffusion model, have been investigated thoroughly before (Bogacz et al., 2006; Simen et al., 2006, 2009; Balci et al., 2011). Specifically, it has been shown that this function is uni-modal in the whole parameter space. Our simulations, not reported here, showed that this is also the case for the independent race model used here.

**Table 2:** **Task parameters used in the first example**.

**Parameter**	μ_1_	μ_2_	σ	*T*^*ND*^	*r*^*C*^	*r*^*I*^	*T*^*DP*^
**Value**	0.1	0	0.05	0.5	5	−5	5

**Figure 4:**
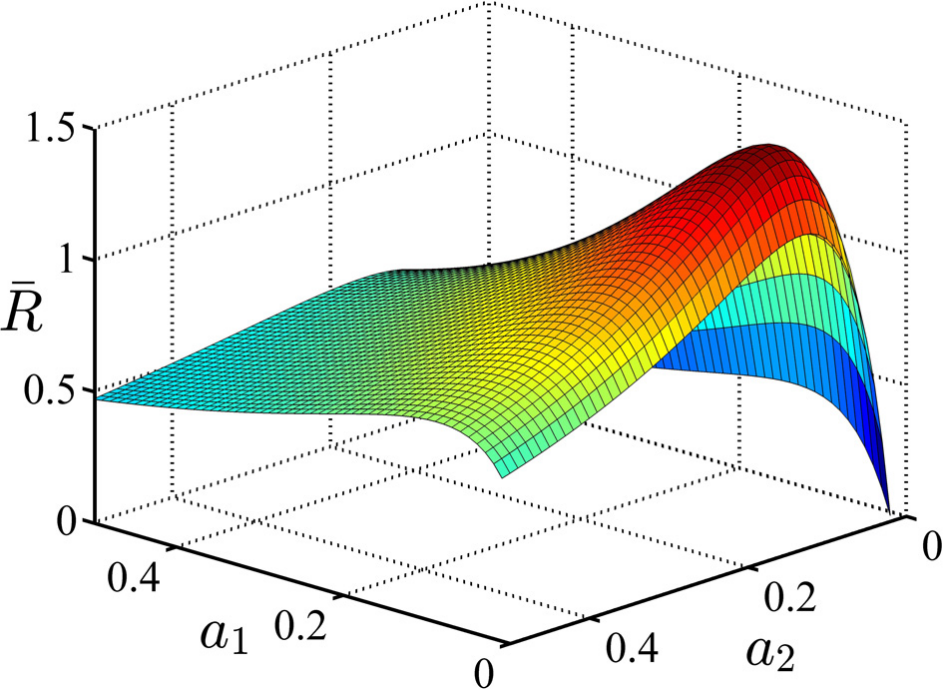
**The average reward rate in example 2**. In this example, there are two conditions and each trials starts with presentation of a cue associated with these conditions. It is assumed that the subject sets different decision thresholds for each condition. In this figure, the average reward rate is plotted as a function of these decision thresholds. *a*_1_ denotes the decision threshold in condition 1 and *a*_2_ denotes the decision threshold in condition 2. The task parameters are given in Table 3.

In the second example, we consider an experiment similar to what was shown in Figure 1. In this experiment, each trial could come from one of the two conditions with equal probability. The task parameters are given in Table 3. In this table, μ^*j*^_*i*_ is the drift coefficient of accumulator *j* in condition *i*. As explained before, in this experiment the subject can set separate decision thresholds for different conditions. The average reward rate as a function of the decision threshold in condition 1, *a*_1_, and condition 2, *a*_2_, is shown in Figure 4. Although we do not prove it here, our simulations suggest that this function is also uni-modal over the whole parameter space and so there is one pair of the decision thresholds that maximize it. In Figure 4, the average reward rate is maximized when *a*_1_ = 0.06 and *a*_2_ = 0.11. As can be seen in Table 3, in both conditions, the reward for the correct response is *r*^*C*^_*j*_ = 2 but the punishments are different. In condition 2, the punishment is greater and because of that, the subject might ponder more in those trials and so the optimal decision threshold for condition 2 is greater than condition 1.

**Table 3:** **Task parameters used in the second example**.

**Parameter**	μ^1^_1_	μ^2^_1_	μ^1^_2_	μ^2^_2_	σ	*T*^*ND*^	*r*^*C*^_1_	*r*^*I*^_1_	*r*^*C*^_2_	*r*^*I*^_2_	*T*^*DP*^	*T*^*RSI*^
**Value**	0.1	0	0.15	0	0.07	1	2	−1	2	−5	2	1

**Figure 5:**
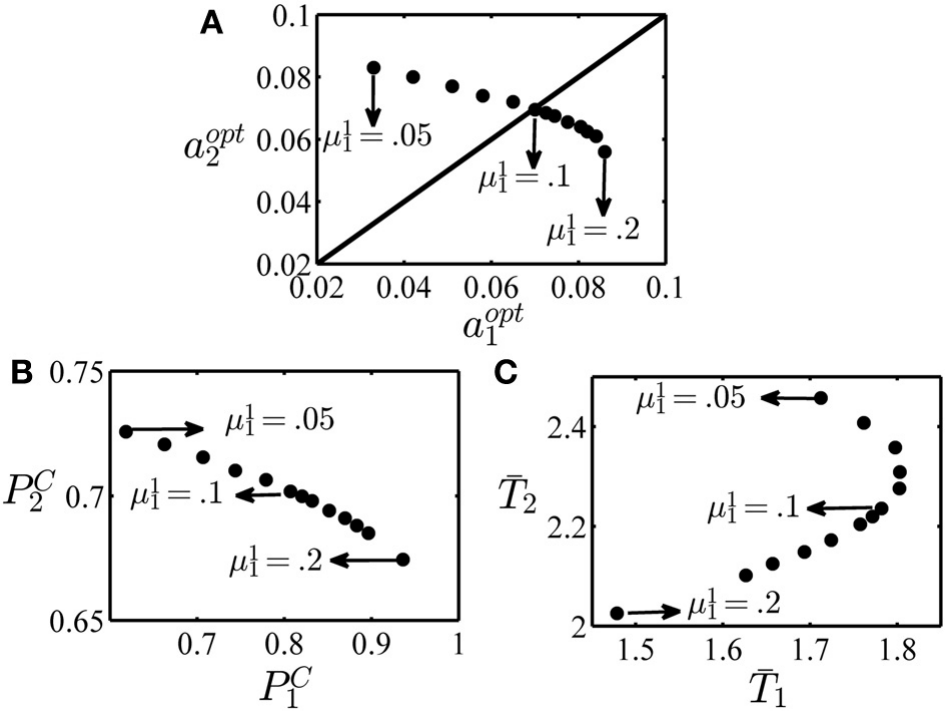
**Optimal decision thresholds, probability of correct and mean reaction times in example 3**. **(A)** The optimal value of the decision threshold in condition 1, *a*^*opt*^_1_, and condition 2, *a*^*opt*^_2_, for different values of the parameter μ^1^_1_ in the interval [0.05, 0.2]. **(B)** The probability of a correct response in condition 1 (denoted as *P*^*C*^_1_) versus condition 2 (denoted as *P*^*C*^_2_). **(C)** The mean time spent on condition 1 (*T*_1_) and condition 2 (*T*_2_). In all figures, the points corresponding to μ^1^_1_ = 0.05, 0.1 and 0.2 are specified by arrows.

In the last example of this section, we examine how the optimal decision threshold in each condition varies when the difficulty of one of the conditions changes. Again, we consider an experiment with two conditions. The task parameters are given in Table 4. As can be seen in this table, all parameters of the two conditions are the same except the reward after correct responses which is 1 in condition 1 and 5 in condition 2. Here, we want to see how the optimal values of the decision thresholds change when the salience level of condition 1, μ^1^_1_, changes. The optimal value of the decision thresholds for several values of μ^1^_1_ in the interval [0.05, 0.2] is plotted in the top panel of Figure 5. When the salience levels in the two conditions are equal, that is μ^1^_1_ = μ^1^_2_ = 0.05 and μ^2^_1_ = μ^2^_2_ = 0, the optimal value of the decision threshold in condition 2 is larger than condition 1 (*a*^*opt*^_1_ = 0.033 and *a*^*opt*^_2_ = 0.083). This is because each correct response in condition 2 leads to higher value of reward and so it is worth it to set a higher decision threshold for this condition and so on average spent more time on this condition than condition 1 and make more correct responses. However, as the salience level of condition 1 increases and this condition becomes easier than condition 2, the optimal decision threshold in condition 1 increases while it decreases for condition 2. To investigate this situation more, the probability of giving a correct response and mean time spent in each condition when the optimal decision thresholds are recruited are shown in the left and right panels at the bottom of Figure 5, respectively. As seen, by increasing μ^1^_1_, the optimal decision thresholds change in a way that the probability of correct response increases for condition 1 and decreases for condition 2. The mean time spent in each condition shows a more complicated pattern. In conclusion, even when the task parameters of only one condition change, the subject should adjust her speed and accuracy in all conditions to maximize the global average reward rate.

**Table 4:** **Task parameters used in the third example**.

**Parameter**	μ^2^_1_	μ^1^_2_	μ^2^_2_	σ	*T*^*ND*^	*r*^*C*^_1_	*r*^*I*^_1_	*r*^*C*^_2_	*r*^*I*^_2_	*T*^*DP*^	*T*^*RSI*^
**Value**	0	0.05	0	0.07	0.5	1	−2	5	−2	2	0.5

## 4. A stochastic process model of the experiment

The main aim of this paper is to propose a computational model of how subjects learn the optimal decision thresholds in the experimental design explained before. The proposed model is based on well-known reinforcement learning algorithms previously used to model optimal action selection and decision making in animals and humans (Barto, 1995; Montague et al., 1996; Schultz et al., 1997; Sutton and Barto, 1998). In these algorithms, the learning problem is formulated as an optimal control problem in a stochastic environment. One critical step in modeling in this framework is to specify the environment corresponding to the problem in hand. In this section, we show how our experimental design can be modeled as a stochastic process called a semi-Markov decision process. In what follows, we first explain Markov decision processes and then discuss how semi-Markov decision processes generalize them to continuous time problems. Finally, we show how our problem can be cast as a semi-Markov decision process.

### 4.1. Markov decision process

In a Markov decision process an agent (e.g. an animal or a robot) is interacting with a stochastic environment. The environment consists of *N* states *S* = {*s*^1^, …, *s*^*N*^} and at each time step *k* it is in one of these states, say *s*_*k*_ = *s*^*i*^. At each time step, the agent can choose an action from the set of *M* possible actions *A* = {*a*^1^, …, *a*^*M*^}. After taking action *a*_*k*_ the environment transfers to a new state *s*_*k* + 1_ with probability **T**^*u*^_*ij*_ (*k*) = Pr(*s*_*k* + 1_ = *s*^*j*^|*s*_*k*_ = *s*^*i*^, *a*_*k*_ = *a*^*u*^) and the agent receives a probabilistic reward *r*_*k*_ = *r* with probability **R**^*u*^_*ij*_ (*r*, *k*) = Pr(*r*_*k*_ = *r*|*s*_*k*_ = *s*^*i*^, *s*_*k* + 1_ = *s*^*j*^, *a*_*k*_ = *a*^*u*^). The important aspect of these functions is that they possess the Markov property. That is, the transition probability **T**^*u*^_*ij*_ and reward probability **R**^*u*^_*ij*_ only depend on the state at time *k* and the action *a*_*k*_ and not the whole history of states and actions {*s*^1^, *a*^1^, …, *s*^*k*^, *a*^*k*^}. More formally:

(3) $$\mathrm{Pr}\left(s_{k+1}=s^{j}|s_{k},a_{k},\cdots ,s_{1},a_{1}\right)=\mathrm{Pr}\left(s_{k+1}=s^{j}|s_{k},a_{k}\right)$$

(4) $$\mathrm{Pr}\left(r_{k}=r\text{|}s_{k+1},s_{k},a_{k},\cdots ,s_{1},a_{1}\right)=\mathrm{Pr}\left(r_{k}=r\text{|}s_{k+1},s_{k},a_{k}\right)$$

This structure is called a Markov decision process (MDP). In short, an MDP consists of a 4-tuple 〈*S*, *A*, **T**, **R**〉 such that **T** and **R** possess the Markov property. The state and action spaces in an MDP can be continuous.

The agent's goal in an MDP is to find the optimal policy. A policy π : *S* × *A* → [0, 1] is a function that maps a state-action pair (*s*, *a*) to the probability of selecting action *a* in state *s*. To define the optimal policy we need a notion of optimality. This notion can be formalized based on the agent's desire to maximize a function of received rewards called *return*. One popular form of the return function used in many applications of reinforcement learning is *expected discounted sum of future reward*:

(5) $$\text{E}\left[\sum_{k=0}^{\infty }\gamma^{k}r_{k}\right]$$

where the operator E denotes expectation over all trials. The parameter γ is called the discounting factor and determines the relative weighting of immediate versus later rewards. The optimal policy will maximize this return. One reason for popularity of this return in the literature of reinforcement learning is that, as we will see in section 5.1, it will lead to a set of recursive equations for finding the optimal policy. Some of the psychologically more plausible returns (e.g., hyperbolic discounting) do not possess this property (for a fuller discussion see Daw, 2003, section 2.1.4).

Based on this notion of return, the value of state *s*^*i*^ at time step *k* under the policy π is defined as:

(6) $$V_{\pi }\left(s^{i},k\right)=\text{E}\left[\sum_{j=k}^{\infty }\gamma^{j-k}r_{j}\text{|}s_{k}=s^{i},\pi \right]$$

This function is called the *state value function* and is the expected discounted sum of rewards that the agent expect to receive given that the state at time step *k* is *s*_*k*_ = *s*^*i*^ and the agent will choose actions based on policy π afterwards. It is easy to show that an optimal policy that maximizes the return 6 will also maximize the state value functions for all time steps and states. Thus a policy π^*^ is optimal if:

(7) $$V_{\pi^{*}}\left(s,k\right)\geq V_{\pi }\left(s,k\right)\;for\;all\;s\in S\;and\;k\;and\;all\;policies\;\pi$$

that is, if the state value functions under that policy are greater than those under any other policy.

### 4.2. Semi-markov decision process

In an MDP the state transitions occur at discrete time steps. Semi-Markov decision processes (SMDPs), generalize MDPs by allowing the state transitions to occur in continuous irregular times. In this framework, after the agent takes action *a* in state *s*, the environment will remain in state *s* for time *d* and then transits to the next state and the agent receives the reward *r*. The dwell time *d* is a random variable with probability density *D*(d;*s*, *a*). An SMDP is specified by the 5-tuple 〈*S*, *A*, **T**, **R**, *D*〉 where **T**, **R** and *D* possess the Markov property. This process is called semi-Markov because the transition from one state to another not only depends on the current state and action but also on the time elapsed since the action has been taken.

Since *D* is a function of action *a*, the dwell time in each state depends on the agent's policy. This means that in an SMDP, in addition to the total reward, the total time to achieve that reward depends on the policy. Thus, it is reasonable to define the optimal policy based on a return that takes both reward and dwell time into account. This makes the average reward rate an appealing choice for the return in an SMDP. Assuming that the rewards are delivered only after each transition (and not during the dwell time) the average reward rate of an SMDP starting at state *s*^*i*^ under the policy π is defined as follows (Das et al., 1999):

(8) $${\bar{R}}^{\pi }\left(s^{i}\right)=\underset{N\to \infty }{\lim}\frac{\text{E}\left[\sum_{k=0}^{N}r_{k}|s_{0}=s^{i},\pi \right]}{\text{E}\left[\sum_{k=0}^{N}d_{k}|s_{0}=s^{i},\pi \;\right]}$$

The state value functions are defined accordingly. The optimal policy of an SMDP, then, maximizes the average reward rate.

### 4.3. An SMDP model of the experiment

**Figure 6:**
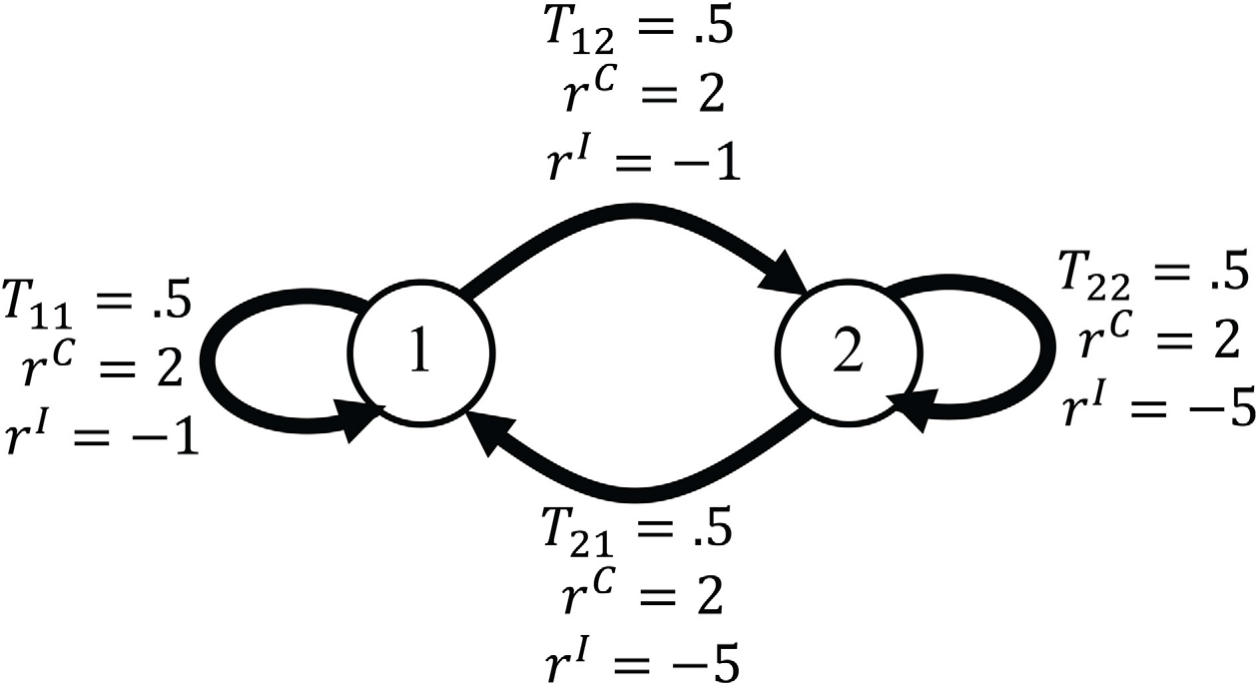
**The SMDP corresponding to the second example in section 3.3**. In the example each trial could come from one of two conditions and so the corresponding SMDP has two states. The states are shown by circles. The arrows show transitions between states. The probability of each transition and the reward that the agent receives after each transition are written on the arrows.

In this section, we show how the experimental design explained in section 2 can be modeled as an SMDP. Each SMDP is specified by the five-tuple 〈*S*, *A*, **T**, **R**, *D*〉 and so to explain our model we should show how these functions correspond to different components of the experiment and mechanisms of subjects' decision making. Before formally defining each of the components of the model, we explain them using the examples in section 3.3. The SMDP corresponding to the second example in section 3.3 is shown in Figure 6. There are two conditions in this example. In our model, we assume that each condition corresponds to one state of the environment and so for this example the corresponding SMDP has two states. After presentation of each cue at the beginning of each trial, the environment transits to one of these states. The dwell time in an SMDP is defined as the time between the transition from one state to another. Since the state transitions in the model occur at the beginning of each trial (by the presentation of the cue), the dwell time is the time between the presentation of a cue in one trial and the time of the presentation of the cue in the next trial. The critical aspect of the model is the way that we define actions in the corresponding SMDP. In our experimental design, the main concern is the relationship between the decision threshold in each condition and the average reward rate. On the other hand, in an SMDP in each state the agent tries to take actions that maximize the average reward rate. This suggests a plausible choice for actions in the corresponding SMDP: the action in each state is the decision threshold of the information accumulators. In this way, by learning the optimal policy in the SMDP, the subject is actually learning the optimal value of the decision threshold for each condition. The decision threshold affects both the reaction time and the accuracy and so in the corresponding SMDP, actions affect both the reward probabilities and the dwell times. Since the decision thresholds can be any positive value, the action space in each state is the continuous space of all positive numbers.

The transition from one state to another is determined by the probability that a trial comes from a specific condition. In the example we are considering here, this probability is 0.5 for each condition. The reward that subject receives after each correct and incorrect response depends on the condition. In Figure 6, these quantities are shown on the arrows that indicate transitions between states.

Based on this description, the functions 〈*S*, *A*, **T**, **R**, *D*〉 can be specified as follows:

*The state space S:* the state space is the discrete set of all possible conditions in the experiment, that is *S* = {*C*_1_, …, *C*_*N*_*c*__}.

*The transition probability function* **T**: For sake of simplicity, in this paper we only consider experiments in which the probability of each trial coming from a specific condition does not depend on either subject's response or the condition presented in the previous trial. As we explained, this probability is instead determined by the experimenter. Thus, the transition probability function is defined as follows:

(9) $$T_{ij}^{u}(k)=\mathrm{Pr}\left(s_{k+1}=C_{j}\text{|}s_{k}=C_{i},a_{k}=a^{u}\right)=\mathrm{Pr}\left(s_{k+1}=C_{j}\right)=P_{j}$$

where *C*_*j*_ denotes the *j*^th^ condition which corresponds to the *j*^th^ state of the environment.

*The reward probability function* **R**: As it was explained before, the reward that subject receives after responding in each trial, depends on the condition and the subject's response. Specifically, in condition *i* the subject receives reward *r*^*C*^_*i*_ for each correct response and *r*^*I*^_*i*_ for each incorrect response. Therefore, the probability of receiving a reward *r* in each condition depends on subject's accuracy and so her decision threshold in that condition. Formally, the reward probability function is defined as follows:

(10) $$\begin{array}{l} R_{ij}^{u}(r,k)=\mathrm{Pr}\left(r_{k}=r\text{|}s_{k}=C_{i},s_{k+1}=C_{j},a_{k}=a^{u}\right)\\ \;=\{\begin{array}{cc} \begin{array}{c} P_{i}^{C} \end{array} & if\;r=r_{i}^{C},\\ 1-P_{i}^{C} & if\;r=r_{i}^{I},\\ 0 & otherwise \end{array} \end{array}$$

*The dwell time probability density function *D*:* The dwell time in an SMDP is the time that it takes between transition from one state to another. In our model, this time is the sum of four parts: non-decision time, response time, delay penalty and RSI. The response time is the time between the presentation of the stimulus and the time that the first accumulator hits its decision threshold. The delay penalty depends on the subject's response and the trial condition. The probability density of the dwell time for each condition is a function of the subject's decision threshold and the task parameters in that condition. The mean dwell time in condition *i* is:

(11) $$\text{E}[d]={\bar{T}}_{i}^{C}\cdot P_{i}^{C}+\left({\bar{T}}_{i}^{I}+T_{i}^{DP}\right)\cdot \left(1-P_{i}^{C}\right)+T_{i}^{RSI}+T^{ND}$$

*The action space A:* As it was explained above, the action space in each state is the space of all positive real numbers. The policy π(*s*_*k*_ = *C*_*i*_, *a*_*k*_ = *a*) is the probability density function that specifies the likelihood of setting the value *a* as the decision threshold when the cue associated with condition *C*_*i*_ is presented.

## 5. Model

So far, we have shown how our experimental design can be modeled as an SMDP. Following Gold and Shadlen (2002) we speculated that a rational subject learns to balance her speed and accuracy in each condition such that the average reward rate is maximized. The question, then, is how the subject learns this optimal behavior. In this section, we propose a normative model of learning the optimal SAT in our experimental design. In the SMDP framework proposed above, the problem of optimal SAT is equivalent to the problem of learning the optimal policy that maximizes the average reward rate. Fortunately, the problem of learning the optimal policy in an MDP and SMDP has been investigated thoroughly in the machine learning and computer science literature. Specifically, the reinforcement learning (RL) algorithms provide a mechanism for learning the optimal policy without any knowledge about the dynamic of the environment and only by experiencing it (Bertsekas and Tsitsiklis, 1996; Sutton and Barto, 1998). In an SMDP, the dynamic of the environment is determined by the functions **R**, **T** and *D*. The RL algorithms assume that the agent does not know these functions and can only observe noisy samples from them. This property makes these algorithms appropriate for our problem: here, the subject can only observe the reward, reaction time and condition in each trial and she should learn the optimal value of decision threshold based on these observations. Another appealing feature of these algorithms is that they provide a biologically plausible account of learning. It has been shown that the pattern of fluctuations in the firing of dopaminergic neurons in ventral tegmental area and surrounding neurons in tasks that involve prediction of reward, resembles a signal called the *temporal difference error*, which plays a central role in the RL algorithms (Montague et al., 1996; Schultz et al., 1997).

In this section, we first explain the temporal difference learning method and then propose a model that uses this method to solve the optimal SAT problem.

### 5.1. Temporal difference learning

As we explained before, the optimal policy in MDPs and SMDPs is defined as the policy that maximizes the state values for all states (see inequality 7). Therefore, to find the optimal policy a learning algorithm should be able to compute the state values for a given policy. For return function 5, the state values are defined in Equation 6. This equation can be written in a recursive form:

(12) $$V_{\pi }(s_{k})=\text{E}\left[r_{k}+\gamma V_{\pi }\left(s_{k+1}\right)\right]$$

This equation is known as the Bellman equation. The expectation on the right hand side of this equation is taken with respect to all possible actions and states and so depends on the functions **T** and **R**. Notice that the Bellman equation provides one equation for each state in the state space and so can be considered as a system of equations. If the functions **T** and **R** are known, dynamic programming methods can be used to solve this system of equations efficiently (Bertsekas and Tsitsiklis, 1996). However, in many situations (including our problem) the agent does not know these functions. The temporal difference (TD) leaning method provides a simple and efficient solution to this problem. In its simplest form, the TD learning method uses an estimate of the difference between the two sides of Bellman equation 12 to learn the state value functions. This estimate is called the temporal difference error and is defined as follows:

(13) $$\delta_{k}=r_{k}+\gamma {\hat{V}}_{\pi }^{k}(s^{j})-{\hat{V}}_{\pi }^{k}(s^{i})$$

where ${\hat{V}}_{\pi }^{k}(s)$ is the agent's estimate of the value of state *s* at time step *k*, *s*^*i*^ and *s*^*j*^ are the state of the environment at steps *k* and *k* + 1 and *r*_*k*_ is the one step reward that the agent earned by going from *s*^*i*^ to *s*^*j*^. The agent then updates its estimate of the value of state *s*^*i*^ using this error signal:

(14) $${\hat{V}}_{\pi }^{k+1}(s^{i})={\hat{V}}_{\pi }^{k}(s^{i})+\alpha_{c}.\delta_{k}$$

where α_*c*_ is the learning rate.

Das et al. (1999) showed that for an SMDP with the average reward rate return defined in Equation 8, the TD error signal should be computed as follows:

(15) $$\delta_{k}=r_{k}-{\hat{\rho }}_{k}\cdot d_{k}+{\hat{V}}_{\pi }^{k}(s^{j})-{\hat{V}}_{\pi }^{k}(s^{i})$$

where ${\hat{\rho }}_{k}$ is an estimate of the average reward rate defined in Equation 8 at time step *k* (in the next section, we explain how this estimate can be computed).

Equation 15 together with the update Equation 14 provide an algorithm for learning the state values for a given policy π in an SMDP. However, they do not specify how the optimal policy can be learned. In the next section, we explain a method for learning the optimal policy based on the TD learning algorithm.

### 5.2. A model of learning optimal decision thresholds

**Figure 7:**
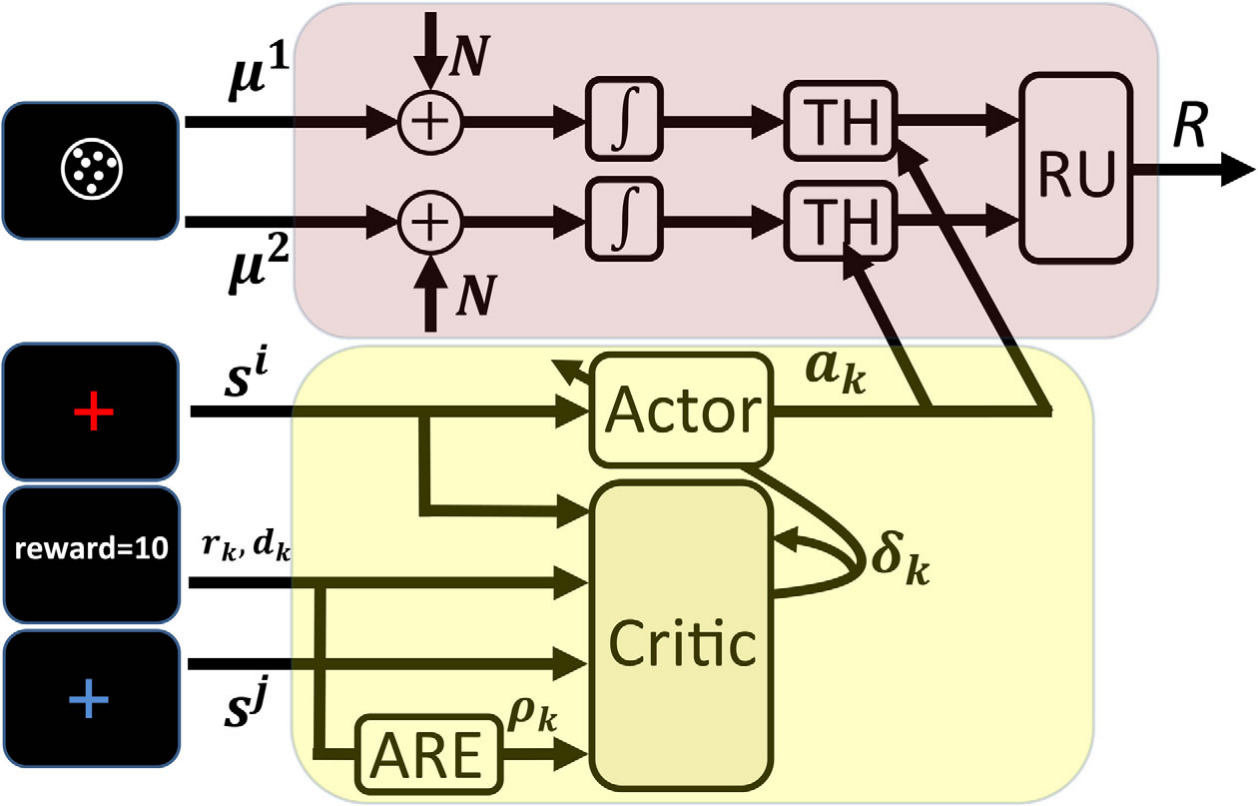
**Schematics of the proposed model of learning the optimal decision thresholds**. All frames of a trial in Figure 1 are shown here as the input to different components of the model. The cue presented at the beginning of the trial (the red cross-hair here) determines the current state, *s*_*k*_, and acts as the input to the actor. Based on *s*_*k*_ and the current policy the actor chooses a value *a* for the decision thresholds of the information accumulators. The arrows from the output of the actor to the threshold units in the accumulators (denoted as TH in the figure) show that the decision thresholds are set by the actor. The noisy information accumulation is represented in the figure by two channels in which the noise *N* is added to the signals μ^*i*^ and passed through integrators. This noisy accumulated information is sent to the threshold units and finally to the response unit (RU in the figure) that determines which accumulator has finished processing first. After responding, the model receives the feedback which is the inputs to the critic. Other inputs of the critic are the cue presented in the next trial (which determines the next state *s*_*k* + 1_) and the estimate of the average reward rate. The ARE unit in the figure receives the reward as its input and computes an estimate of the average reward rate using equation 19. Finally, the critic computes the TD error signal through equation 17 and uses it to update both the estimate of state values and the policy.

In this section, we propose a model for learning the optimal decision thresholds. This model is based on the TD learning algorithm. The schematics of the model is shown in Figure 7. The frames shown on the left of this figure as the inputs to different parts of the model, are exactly those that were shown in Figure 1 (these are the frames shown in one trial of the task).

The model consists of two units: an *information accumulation unit* and an *actor-critic unit*. The information accumulation unit is responsible for processing the stimulus and selecting the appropriate response in each trial. The stimulus presented in each trial is considered as the input to this unit and the selected response (denoted as *R* in the figure) is the output of the unit. This unit is an independent race model. As we discussed in section 3.2, in the independent race model, the correct and incorrect responses each have a separate accumulator (modeled as a diffusion process) and assumes that whenever the accumulated information reaches one of the thresholds (TH in the figure), the subject will respond. The unit named RU in the figure, simply determines which of the accumulators has won the race and so determines the response.

The speed and accuracy are controlled by the value of the decision threshold in the information accumulation unit. The value of this parameter is set by the other unit of the model, the actor-critic unit (in the figure, the arrows from the output of the actor-critic unit to the threshold units are intended to show this) and so this unit is responsible for learning to solve the optimal SAT problem. Actor-critic architecture is one the most popular TD learning algorithms. Specifically, among several TD based algorithms proposed in the RL literature, the actor-critic algorithm has received significant attention in the computational neuroscience literature. This is because different components of this model mapped nicely to the anatomy of basal ganglia, a brain circuit know to be involved in many motor and cognitive functions (Barto, 1995; Doya, 2000; Frank, 2005, 2006; Bogacz and Larsen, 2011).

The actor-critic architecture, as its name suggests, consists of two units: an actor unit and a critic unit. The actor unit has a representation of the current policy and in each state selects an action based on this policy. In the SMDP model of our experimental design, the state is determined by the cue presented at the beginning of a trial and the action is the value of the decision threshold for that trial. Thus, in our model the cue presented at the beginning of a trial is the input to the actor unit while its output is the decision threshold for that trial (see Figure 7). In each trial, after the presentation of the cue the actor sets the decision thresholds of the information accumulation units. After the presentation of the stimulus, the information accumulation unit selects a response based on which of its accumulators has reached its threshold sooner. Based on the selected response and the trial condition, a reward is presented to the subject and the next trial starts after a while.

At the moment that the cue of the next trial is presented, the critic unit plays its role. The role of this unit, as its name implies, is to criticize the action taken by the actor in a trial. In our model, the critic evaluates if the chosen decision threshold in a trial leads to better or worse than expected performance in that trial. The critic does this by computing the TD error in that trial. To see how the TD error can be employed to evaluate the actions taken by the actor, let us consider an experimental design with only one condition (like the first example in section 3.3). In this situation, *s*^*j*^ = *s*^*i*^ = *C*_1_ and so the terms ${\hat{V}}_{\pi }^{k}(s^{j})$ and ${\hat{V}}_{\pi }^{k}(s^{i})$ in Equation 15 cancel out each other and the TD error is reduced to $\delta_{k}=r_{k}-{\hat{\rho }}_{k}\;\cdot \;d_{k}$. If the TD error δ_*k*_ is positive for a trial, it means that the amount of reward received in that trial, *r*_*k*_, exceeded the cost spent on that trial which is ${\hat{\rho }}_{k}\;\cdot \;d_{k}$. The term ${\hat{\rho }}_{k}\;\cdot \;d_{k}$ is considered as the cost spent on the trial because when the subject is spending time *d*_*k*_ to receive the reward *r*_*k*_, she is actually losing the opportunity of spending this time on other trials that on average lead to ${\hat{\rho }}_{k}$ of reward per unit of time. Thus, if the actor has chosen a value for the decision threshold for a trial and the TD error for that trial is positive, it means that decision threshold has led to better than expected performance. Similarly, negative TD error means worse than expected performance. In actor-critic algorithm, this feature of the TD error is used to improve the policy: if in a trial the actor takes an action that leads to positive TD error, the probability of taking that action next time increases. Similarly, the probability of taking actions that lead to negative TD error decreases. This way the policy is improved (at least probabilistically) after each trial till it finally converges to the optimal policy.

In the general experimental design in which there could be more than one condition, to compute the TD error, the critic needs to have an estimate of the state values. Thus, in addition to improving the policy, the TD error computed by the critic is used to estimate these values using Equation 14 (in Figure 7 the arrow that goes from the output of the critic back to it is intended to show this).

In sum, in our model, after the presentation of the cue in the next trial [which is equivalent to transition to the new state in the corresponding SMDP (see section 4.3)], the critic computes the TD error which is used both to improve the policy and estimate the state values.

To calculate the TD error using Equation 15, the critic needs to know the current state *s*^*i*^, the next state *s*^*j*^, the reward *r*_*k*_, the dwell time *d*_*k*_ and the estimate of the average reward rate ${\hat{\rho }}_{k}$. Thus, in Figure 7 the frames corresponding to the current trial (the red cross-hair), the reward, the next trial (the blue cross-hair) and also the output of the average reward rate estimator unit (ARE in the figure) are shown as the inputs to the critic unit.

So far, we have explained the role of different units of the model in processing the stimulus and selecting response, setting the decision thresholds and improving the policy. To complete the model, three issues should be addressed and the rest of this section is devoted to them: first, how the policy is represented in the actor, second, how the policy can be improved using the TD error and third, how the average reward rate can be estimated.

The first two problems are tightly related and so we explained them first. When the actor-critic algorithm is used in discrete action space problems, the policy can be represented as a probability mass function with one probability value for each action in the action space. When an action is taken by the actor, the probability of taking it in the next trial will be increased or decreased proportional to the TD error, and the probability of taking other actions will be normalized accordingly. However, when the action space is continuous (as is the case in our model), the policy takes the form of a probability density function, and so updating it using the method for discrete action spaces is not feasible anymore. Another problem associated with continuous action spaces is action selection: even if we are able to fully specify the policy π(·), then how should the actions be selected? Several methods have been proposed to address these problems. The comparison between these methods is outside the scope of this paper. Here, we use a slight modification of a simple algorithm proposed by Gullapalli (1990). In this algorithm, the policy is represented by a Gaussian distribution. For a 1-dimensional action space the policy takes the following form:

(16) $$\pi \left(s_{k}=s^{i},a_{k}=a\right)=\frac{1}{\sqrt{2\pi }\cdot \nu_{i}}\cdot \exp\left(-\frac{{\left(a-m_{i}\right)}^{2}}{2{\left(\nu_{i}\right)}^{2}}\right)$$

where π(*s*_*k*_ = *s*^*i*^, *a*_*k*_ = *a*) specifies the likelihood of choosing the value *a* as the action in state *s*^*i*^, and *m*_*i*_ and ν_*i*_ are the mean and standard deviation of the Gaussian distribution representing the policy in state *s*^*i*^. An advantage of representing the policy as a parametric distribution is that during learning, we just need to update the parameters. In other words, the problem of updating the policy reduces to the problem of updating its parameters. The Gaussian distribution in Equation 16 has only two parameters: *m* and ν. Gullapalli (1990) suggested the following updating rule for the parameter *m*:

(17) $$\begin{array}{c} \begin{array}{l} m_{i}(k+1)\leftarrow m_{i}(k)+\alpha_{m}\cdot \Delta m_{i}(k)\\ \;\Delta m_{i}(k)=\delta_{k}\cdot \left(\frac{a_{k}-m_{i}(k)}{{\left(\nu_{i}(k)\right)}^{2}}\right) \end{array} \end{array}$$

In Gullapalli's algorithm ν is also updated but for sake of simplicity we do not consider updating it here (ν remains constant during learning).

In the actor-critic implementation of this algorithm, in each state the actor draws a sample from the distribution Equation 16 and takes it as the action. After receiving reward the critic computes the TD error signal. This signal is then used to update the policy parameter using Equation 17. For appropriate choice of learning rates, this algorithm will eventually converge to the optimal policy.

The parameter ν can be considered as the exploration-exploitation parameter. For small values of this parameter, the Gaussian distribution is highly concentrated around its mean, *m*, and so most of the actions (which are random samples from this distribution) will be close to the mean. In this case, the algorithm cannot explore the action space enough. On the other hand, for large values of ν, many of the actions will be exploratory. In this case, even if the algorithm finds the optimal value of *m*, many of the selected actions will still be suboptimal. One way to balance between exploration and exploitation is to start the algorithm with large values of ν and decrease its value gradually during learning.

Now, we turn to the third problem mentioned above. In Equation 15, ${\hat{\rho }}_{k}$ is the estimate of the global average reward rate. This signal is estimated by a linear filter named ARE in Figure 7. Before explaining how this unit works, we should clarify a point. Both the actor and the critic units work at discrete time steps. Specifically, although we can implement them as continuous time systems, they only do their computations at either the beginning or at the end of a trial. Therefore, all signals computed in these two units are indexed by the discrete index *k*. However, to estimate the average reward rate, the ARE unit needs to work in continuous time and so its input and output are functions of time *t*. Specifically, the input to this unit is the signal *U*(*t*) = ∑_*k*_ *r*_*k*_ · δ(*t* − *t*_*k*_) where δ(·) is the Dirac delta function, *r*_*k*_ is the reward received in the *k*^th^ trial and *t*_*k*_ is the time at which this reward was received. It is assumed that the rewards are delivered at the time of state transitions and so ${\hat{V}}_{\pi }^{k}(s^{i})$ with *d*_*j*_ being the dwell time in the *j*^th^ trial. The signal *U*(*t*) is the train of impulses created by delivery of rewards. The ARE unit acts as a linear filter on its input and so its output is computed as follows:

(18) $$\frac{d\hat{\rho }(t)}{dt}=-\alpha_{\hat{\rho }}\cdot \hat{\rho }(t)+U(t)$$

The signal $\delta_{k}=r_{k}-{\hat{\rho }}_{k}\;\cdot \;d_{k}$ is the estimate of the average reward rate at time *t*. To compute the TD error, the critic uses the value of this signal at the end of each trial, that is at times *t*_*k*_. It is easy to show that:

(19) $${\hat{\rho }}_{k}=\hat{\rho }(t_{k})=\left({\hat{\rho }}_{k-1}+\alpha_{\hat{\rho }}\cdot r_{k-1}\right)\cdot e^{-\alpha_{\hat{\rho }}\cdot d_{k}}$$

In sum, in one trial of the experiment different components of the model work in the following way: after the presentation of the cue, the actor draws a random sample from the Gaussian distribution in Eqaution 16 and sets the thresholds of the accumulators equal to this value. After the presentation of the stimulus, the accumulators race till one of them reaches its threshold and selects a response. Based on the response and the trial condition a reward is delivered and the next trial starts. After the presentation of the cue at the beginning of the next trial, the critic computes the TD error using Equation 15. This error signal is then used to update both the estimate of state values (using Equation 14) and the policy (using Equation 17). The actor then selects a new threshold for the new trial based on the presented cue.

## 6. Simulation results

In this section, we analyze the performance of the proposed model in two simulations. The first simulation corresponds to the first example given in section 3.3 in which no cue is presented at the beginning of trials and so there is only one state in the corresponding SMDP. The second simulation corresponds to the second example in that section in which there are two trial conditions and each of them is associated with a specific cue presented at the beginning of each trial and so the corresponding SMDP has two states.

### 6.1. Simulation 1: one condition with no cue

In the experimental design considered in this simulation, no cue is presented at the beginning of trials. There could be one or more than one conditions in the task but all conditions are intermixed and the subject does not know the number of conditions in the task. As explained before, because there is no cue, the subject will treat all the conditions the same and so even if there are more than one condition she will set one decision threshold for all trials. For this simulation, we use the parameters values given in Table 2 with *T*^*RSI*^ = 0.

**Figure 8:**
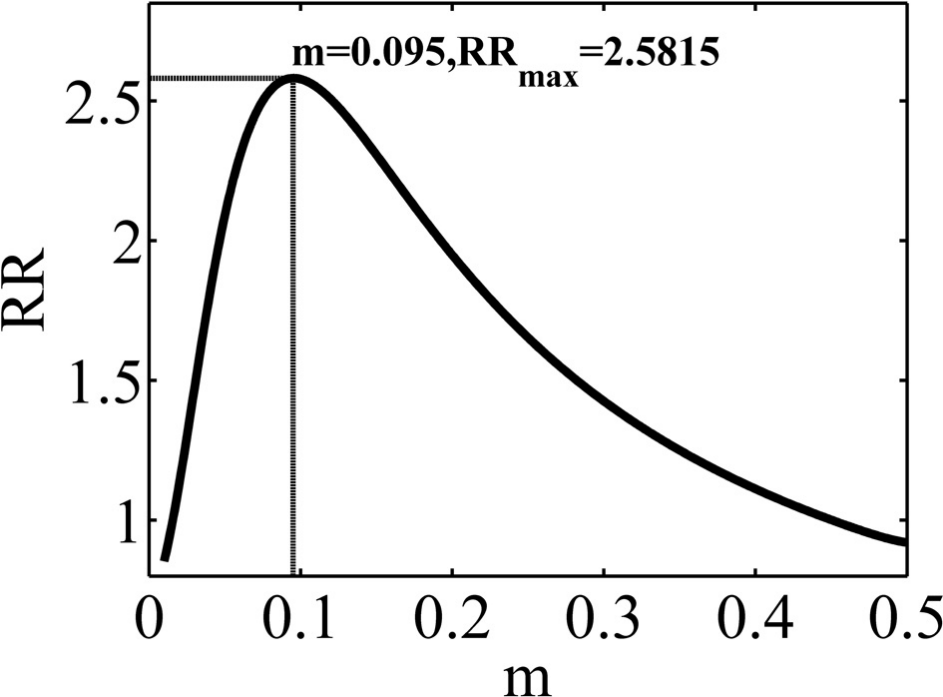
**The average reward rate in the experiment considered in simulation 1, as a function of *m*, the mean of the Gaussian policy**. This function is computed using the method described in Supplementary Material.

We use the Gaussian policy specified in Equation 16 with ν^2^ = 2.25 × 10^−4^. All other parameters being fixed, the average reward rate will be only a function of the mean of the Gaussian, *m*. The average reward rate as a function of *m* is shown in Figure 8. Supplementary Material explains how this function is computed.

The maximum reward rate is equal to *RR*_*max*_ = 2.5812 which is obtained when *m* = 0.095. Here, we investigate the performance of the model in learning this optimal value of *m*.

**Figure 9:**
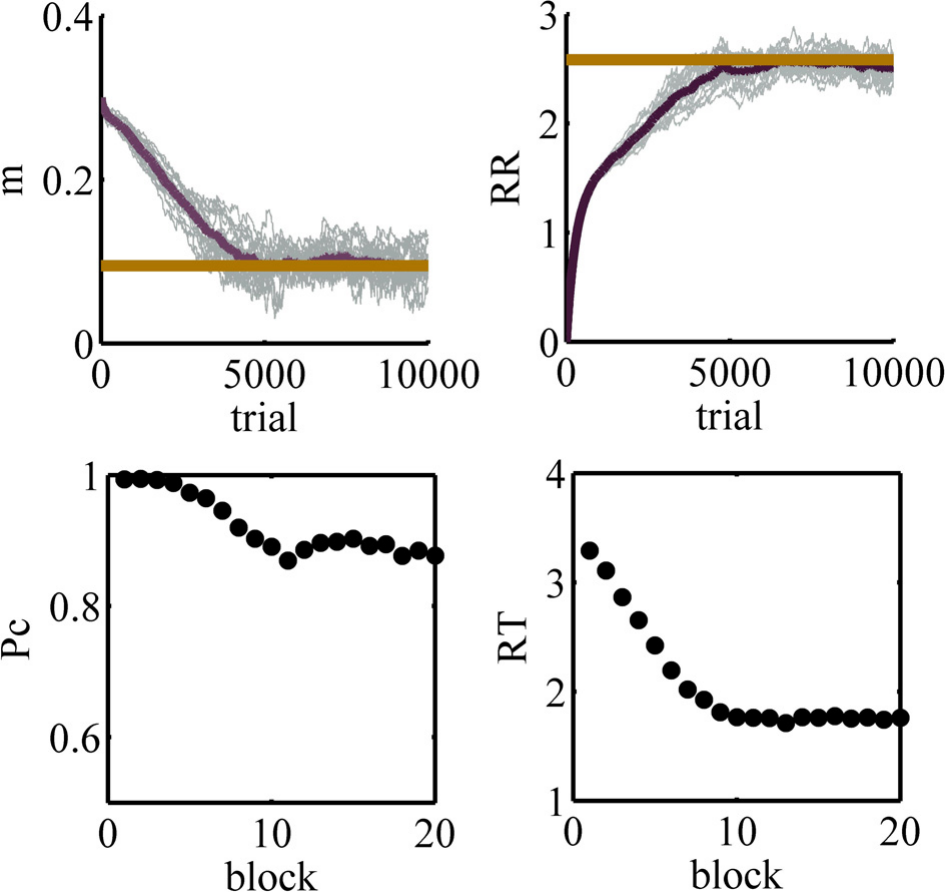
**Results of simulation 1**. **Top left:** the value of the parameter *m* as a function of trial number during learning. **Top right:** the estimated average reward rate, ${\hat{\rho }}_{k}$, as a function of trial. In the top panels, the tick red line shows the optimal value. **Bottom left:** the accuracy as a function of block. **Bottom right:** the mean reaction time as a function of block. In the bottom panels, a block is defined as 500 trials. As learning progresses, the mean of the policy decreases and so the subject chooses lower decision thresholds more often. This leads to lower accuracy but faster responses.

The results of 20 simulations of the model are captured in Figure 9. The learning rates for these simulations are: α_*m*_ = 0.0002, $\alpha_{\hat{\rho }}=0.001$. In the top panels of this figure, thin gray lines correspond to the performance in different simulations and the dark thick line is the average among all simulations. The thick red lines show the optimal values. The left top panel shows the value of the parameter *m* as a function of trial and the right top panel shows the estimated average reward rate (${\hat{\rho }}_{k}$ in Equation 19) as a function of trial. The left and right bottom panels show the accuracy and the mean reaction time averaged over all 20 simulations, respectively, as functions of block number where a block is defined arbitrarily as 500 trials.

At the beginning of learning, the value of the parameter *m* is high and so high values of the decision threshold are chosen more often. Thus, at this stage of learning the accuracy is high and the mean reaction times are also high. In other words, the model is too much conservative and so it cannot achieve the maximum average reward rate. Throughout learning, the model gradually learns to lower the value of the parameter *m*. Finally, at about trial 5800 or so the model finds the optimal value of this parameter. As can be seen in the right top panel, the initial estimation of average reward rate is zero and during learning it approaches the optimal value and finally asymptotes at the optimal value.

The learning of the model may seem slow. This raises the question of whether human subjects are also so slow or if they can find the optimal threshold faster. In a recent study, Balci et al. (2011) investigated human subjects' performance in an experimental design similar to what was used in simulation 1. Their results show that, on average, subjects achieve the optimal performance after about 10 sessions of training (Figures 3a and 8 of Balci et al., 2011). Thus, the learning speed of our model is close to human subjects. Then, the next question is why both the algorithm and the subjects learn slowly. The main reason for this slow learning is a high amount of noise in the function that the agent is trying to maximize. For a single value of the decision threshold, the variance of the reaction time could be high. Also, the accuracy could be a value significantly less than one. Therefore, even if the subject keeps her threshold at a fixed value for several trials, the samples of the average reward rate obtained in each trial would be very noisy and it takes a long time before the subject can achieve a reliable estimate of it.

### 6.2. Simulation 2: two conditions with cue

**Figure 10:**
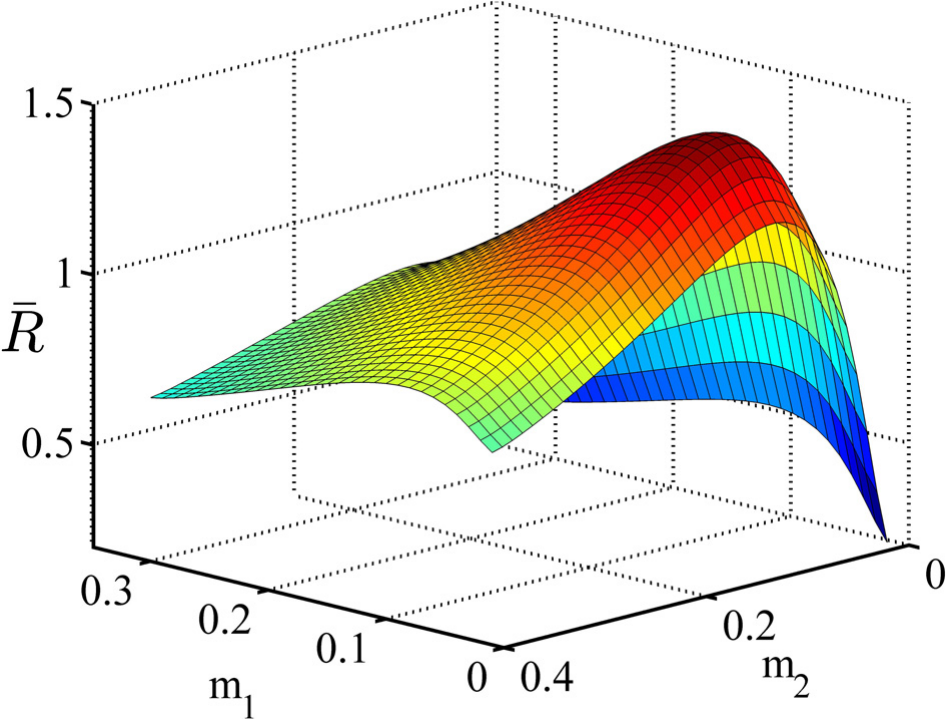
**The average reward rate as a function of the mean of the Gaussian policy in state 1,*m*_1_, and in state 2, *m*_2_**. This function is computed using the method described in Supplementary Materials.

In this simulation, we analyze the performance of the model in the experimental design explained in example 2 of section 3.3. We used the parameters given in Table 3. The policy in each state is represented by a Gaussian distribution with ν^2^_1_ = ν^2^_2_ = 0.015. The average reward rate as a function of the means of these distributions is shown in Figure 10. We used the method explained in Supplementary Material to plot this function. As we see in this figure, the maximum reward rate equals *RR*_*max*_ = 1.43 which is obtained when *m*_1_ = 0.07 and *m*_2_ = 0.12.

The model was simulated in this task for 20 times. The learning rate parameters in the model were α_*m*_ = 0.0005, α_*c*_ = 0.075 and $\alpha_{\hat{\rho }}=0.001$. We have chosen a larger learning rate for the critic than the actor to make sure that the critic learns faster. This is because the critic provides the TD error signal necessary for updating both the critic and the actor and so the policy should not be updated a lot before the critic learns the state values.

**Figure 11:**
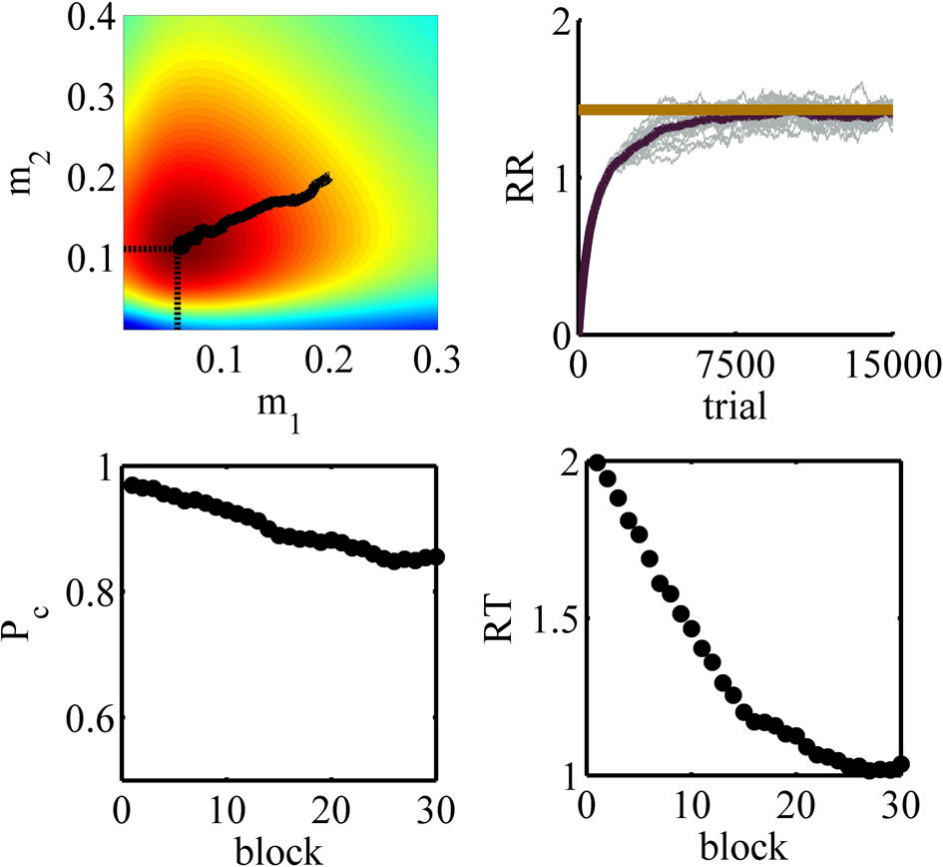
**Results of simulation 2**. **Top left:** the values of the parameters *m*_1_ and *m*_2_ during learning averaged over 20 simulations superimposed on the color map of the average reward rate. The initial point of the path is (*m*_1_(1), *m*_2_(1)) = (0.2, 0.2) and its end point is (*m*_1_(15000), *m*_2_(15000)) = (0.06, 0.11). The optimal value is indicated by the two dash lines. **Top right:** the estimated average reward rate. The optimal value is plotted as a red tick line. **Bottom left:** the accuracy as a function of block number. **Bottom right:** the mean reaction time as a function of block number.

The simulation results are depicted in Figure 11. The panels in this figure correspond to those in Figure 9. The top right panel shows the estimated average reward as a function of trial number. As seen, this function asymptotes in an optimal value after about 8000 trials or so. The top right panel shows the upper view of the average reward rate function shown in Figure 10. The black tick path superimposed on this figure shows the points (*m*_1_ (*k*),*m*_2_ (*k*)) averaged over 20 simulations, with *k* = 1, …, 15000 being the trial number. This curve shows that at the beginning of learning the agent sets the mean of its policy in condition one and two at *m*_1_ = 0.2 and *m*_2_ = 0.2, respectively ((*m*_1_(1), *m*_2_(1)) = (0.2, 0.2) is the starting point of the black path). With extensive learning, the agent learns the optimal value of the thresholds ((*m*_1_(15000), *m*_2_(15000)) = (0.06, 0.11) is the end point of the black path). An interesting point is that this curve shows that on average the learning algorithm takes the shortest path from the starting values to the optimal values of the parameters. Finally, similar to simulation 1, the bottom panels show that the algorithm learns to choose less conservative values of the decision threshold which leads to less accurate but faster responses.

In the previous two simulations, the initial values of the thresholds were higher than the optimal value. We also performed another simulation with the same parameters as simulation 1 but with a lower than optimal initial value of the threshold. Due to space limitation, we do not present the full details of this simulation. It suffices to mention here that the model could learn the optimal value of the threshold in this situation and its performance was at the same level of simulation 1.

## 7. Discussion

In this paper, we suggested a theoretical framework to answer the question of how animals learn to set the decision threshold to maximize the average reward rate. We considered an experimental design in which trials from different conditions are intermixed. A cue associated with each condition is presented at the beginning of each trial and indicates which condition this trial comes from. We derived the expression for the average reward rate in this experiment and investigated the properties of this function and showed that to achieve the optimal average reward rate, the subject has to set different decision thresholds for different conditions. We, then, proposed an SMDP model of the experiment in which each condition is modeled as a state of the environment, decision thresholds are actions and the time spent on each trial is the dwell time in each state. In this way, the problem of learning the optimal decision thresholds becomes the problem of learning the optimal action in each state of the corresponding SMDP. Finally, we proposed a model to solve this problem. In the proposed model, an independent race architecture is responsible for processing the stimulus and selecting responses while an actor-critic architecture learns the optimal value of the decision thresholds.

In the first set of simulation, we considered an experiment in which there is no cue at the beginning of each trial and so there is only one state in the corresponding SMDP. Simen et al. (2006) have proposed a model for learning the optimal decision threshold in this situation. In their model, the decision threshold at time *t* is *a*(*t*) = max Ą(0, *a*_*max*_ − *w* · *r*(*t*)) where *r*(*t*) is the current estimation of the reward rate. To assure that the algorithm converges to the optimal value of *a*(*t*), the two constants *a*_*max*_ and *w* should be chosen such that the line *a*_*max*_ − *w* · *a* passes through the maximum of the function *R*(*a*), the reward rate as a function of threshold *a* (see Figure 3 for an example of this function). Notice that for each set of task parameters the function *R*(*a*) would be different and so different values of *a*_*max*_ and *w* will assure the convergence of the algorithm. In the simulations, the authors assumed that the subjects have learned the optimal values of these parameters through practice under different trial conditions. The model then predicts fast adaptation of the decision threshold for a well-trained subject. The model proposed in this paper explains slow learning of the optimal decision threshold for an untrained subject. Further research is necessary to see how the two approaches can be combined to develop a model of both slow learning of untrained subjects and fast threshold adaptation of well-trained subjects.

In our simulations, we assumed that the drift coefficients remain constant during learning. As a result, when the threshold decreases the accuracy also decreases [see bottom left panels of Figures 9, 11]. However, this may not always be the case. For example, in Balci et al. (2011) the estimated drift coefficient increased with practice. The effect of this increase in drift coefficient and the decrease in the threshold was such that the subjects' accuracy remained constant while the reaction time decreased with practice. It would be interesting to investigate the behavior of the proposed model in this situation and more generally when the task parameters change during learning. It can be imagined, though, that as long as the task parameters do not change very quickly, the model will still be able to learn the optimal thresholds.

The SMDP framework has been utilized previously in the animal learning literature to model the rate of responding in free-operant tasks (Niv, 2007; Niv et al., 2007). A rat placed in an operant chamber can choose to perform one of the several possible actions (nose poking, lever pressing, etc.). In addition, the rat may choose to perform different actions at different rates. Faster responding has the possible benefit of obtaining more reward but it is also associated with higher costs (e.g., energy cost, cognitive load and so on). Niv (2007); Niv et al. (2007) proposed a normative account of how fast each action should be taken to achieve an optimal balance between the benefits of behaving fast and its costs. In this model, taking each action incurs a rate-dependent cost and it is assumed that the rat is trying to maximize the average reward rate. Like Niv's model, the model we proposed in this paper learns to optimally balance between the benefits and costs of acting fast. The benefit of acting fast in both models is to be able to experience more trials. The cost in our framework, however, is having less accuracy. This cost is due to the constraints that the sequential sampling model imposes to the relationship between the speed and accuracy which are in turn imposed by the noise in the stimulus.

One feature of our model is that it suggests a way to integrate the theories of perceptual decision making and reinforcement learning. Traditionally, these theories have been developed separately (see Bogacz and Gurney, 2007 and Bogacz and Larsen, 2011 for a discussion of this matter). Theories of perceptual decision making deal with situations in which the subject should process a noisy stimulus and select the appropriate response based on a known stimulus-response mapping. The reinforcement learning theories, on the other hand, deal with situations in which the stimulus is easily detectable but the subject should learn to take optimal actions in response to each stimulus. In our model, the cue presented in each trial is the easily detectable stimulus for the reinforcement learning (the actor-critic) unit. The role of this unit is to learn the optimal mapping between the cues and the decision thresholds which form the action space in the corresponding SMDP. The noisy stimulus in each trial, on the other hand, is processed by the independent race unit. By this division of labor, the model benefits from the strengths of both sets of theories.

In this respect, our model is in line with the recent effort in integrating these two sets of theories (Bogacz and Gurney, 2007; Dayan and Daw, 2008; Law and Gold, 2009; Rao, 2010; Bogacz and Larsen, 2011; Shenoy and Yu, 2011; Ratcliff and Frank, 2012). Bogacz and Larsen (2011) proposed a computational model of basal ganglia that is capable of learning the optimal stimulus-response mapping when the stimulus is noisy. This model is basically an actor-critic architecture in which the actor is a variant of the sequential sampling models. The critic, crudely speaking, provides the error signal necessary for learning the weights between the sensory units and the information accumulators in the actor. By learning these weights, the model learns the correct stimulus-response mapping. However, because the model is developed in the Markov decision process framework, it cannot solve the problem of optimal balance between speed and accuracy.

Rao (2010) proposed a model in which the perceptual decision making problem was cast as action selection in a partially observable Markov decision process (POMDP). In this model, each stimulus is considered as a state of the environment. The subject, however, does not know the state and instead can only make noisy observations of it at discrete time steps. The subject starts with a prior belief about the state and after each observation she updates her belief using the Bayes rule. At each time step, based on her current belief about the state, the subject can either choose one of the responses or make another observation. Using temporal difference learning, the model learns the optimal mapping between the current belief and these actions. Since the model was not developed to solve the optimal speed-accuracy problem, the cost of time is considered as an arbitrary constant in the model (for example -1 for each time-step that the response is not selected). In contrast, in our model the cost of time is proportional to the average reward rate which in turn depends on the task parameters in all conditions of the task. Further research is needed to compare the two models.

One question that remains open is how the brain performs average reward reinforcement learning. The essential part of this algorithm is the computation of the temporal difference error and this in turn needs an estimate of the average reward rate. Thus, the important question is how the brain estimates the average reward rate. Niv et al. (2007) suggested that the average reward rate is coded as tonic dopamine in the brain. This suggestion is based on the observation that higher levels of tonic dopamine is associated with higher response rate and vice versa (see e.g., Salamone and Correa, 2002). One interesting future line of research, then, would be to use model based fMRI technique to investigate the relationship between the average reward rate signal computed in our model and tonic activity (the activity before the delivery of reward) of brain areas previously associated with reward prediction (e.g., striatum).

### Conflict of interest statement

The authors declare that the research was conducted in the absence of any commercial or financial relationships that could be construed as a potential conflict of interest.
