# Supplementary material for: Learning to maximize reward rate: a model based on semi-Markov decision processes
Source: Front Neurosci. 2014 May 23;8:101. doi: 10.3389/fnins.2014.00101 (PMC4033239; doi:10.3389/fnins.2014.00101)
Supplement: Supplementary file 1 [file DataSheet1.PDF]

Learning to maximize reward rate: a model based on semi-Markov decision processes: Appendix

Arash Khodadadi, Pegah Fakhari and Jerome R. Busemeyer

Appendix A

In this appendix, we derive the formula for the response time and probability of each response in the independent race model. In the model proposed in this paper, the independent race model consists of two accumulators each of which being a diffusion process with fixed decision threshold a. Let T_i denote the first time that the accumulator i passes through its threshold. This time can be define formally as follows:

$$T_i = \inf \{t : X_i(t) \geq a\} \tag{A.1}$$

This time is a random variable and it has been shown that its p.d.f is as follows (see for example (Cox & Miller, 1965)):

$$g_i(t) = \frac{a}{\sigma\sqrt{2\pi t^3}} \exp\left(-\frac{(a-\mu_i t)^2}{2\sigma^2 t}\right) \tag{A.2}$$

which is called an inverse Gaussian distribution. In the race model, the first accumulator that reaches its threshold determines the response and the time at which this happens is consider as the response time. As we explained, in our model accumulator 1 and 2 correspond to correct and incorrect responses, respectively. Therefore, the response would be a correct response if accumulator 1 reaches its threshold before accumulator 2 does so. Let T^C denote the time that this happens. More formally this time can be defined as follows:

$$T^C = \inf \{t : X_1(t) \geq a; X_2(\tau) < a \; for \; all \; \tau < t\} \tag{A.3}$$

It is not hard to show that the p.d.f of this time is as follows:

$$f^C(t) = g_1(t) \cdot (1 - G_2(t)) \tag{A.4}$$

where $G_2(t) = \int_0^t g_2(\tau)d\tau$ is the cumulative distribution function of T_2 and $f^C(t)$ is the p.d.f of the correct responses in the independent race model. The probability of giving a correct response is $P^C = \int_0^\infty f^C(\tau)d\tau$ and the correct mean response time is $\bar{T}^C = \int_0^\infty \tau \cdot f^C(\tau)d\tau$. The formulas for an incorrect response can be derived similarly. If we plug these terms into eq.1, the average reward rate will be a function of the drift and diffusion coefficients and the decision thresholds in different conditions.

Appendix B

In this appendix, we explain how the average reward rate can be computed when the policy is the Gaussian distribution of eq.16. To simplify the argument, let us consider an experiment with one condition (see the first example of section 3.3). In this case, fixing all other parameters, the average reward rate will be a function of the decision threshold a and so can be denoted by $\bar{R}(a)$. For a specific value of the parameters m and ν, the average reward rate should be computed by averaging the function $\bar{R}(a)$ over all values of the parameter a, that is:

$$\bar{R}(m,\nu) = \int_{-\infty}^{\infty} \bar{R}(a)\pi\left(s,a;m,\nu\right) da \tag{B.1}$$

To plot Figure 8, for each value of the parameter m the integral on the right side of this equation was calculated numerically.

Similarly, it can be shown that in the experiments with two conditions (see the second example in section 3.3), the average reward rate can be computed as follows:

$$\bar{R}(m_1,m_2,\nu) = \int_{-\infty}^{\infty}\int_{-\infty}^{\infty} \bar{R}(a_1,a_2)\pi\left(s^1,a_1;m_1,\nu\right)\pi\left(s^2,a_2;m_2,\nu\right) da_1 da_2 \tag{B.2}$$

References

Cox, D. R., & Miller, H. D. (1965). *The theory of stochastic processes.* London: Methuen.
